# A quantile-based composite ionospheric disturbance estimator for RTK positioning reliability

**DOI:** 10.1038/s41598-026-45329-z

**Published:** 2026-03-22

**Authors:** Atis Vallis, Armands Celms, Janis Zvirgzds, Jekabs Vallis

**Affiliations:** 1 Institute of Land Management and Geodesy, Latvia University of Life Sciences and Technologies, Jelgava, Latvia; 2 Faculty of Civil and Mechanical Engineering, Institute of Civil Engineering, Riga, Latvia; 3 Faculty of Science and Technology, University of Latvia, Riga, Latvia

**Keywords:** Ionospheric irregularities, Rate of TEC index (ROTI), TEC gradients, GNSS performance degradation, Quantile-based modeling, RTK risk assessment, Engineering, Mathematics and computing, Physics, Solid Earth sciences

## Abstract

Reliable real-time kinematic (RTK) positioning is highly sensitive to short-term ionospheric irregularities and spatial electron density gradients, which may degrade ambiguity resolution and positioning integrity. Existing disturbance indicators typically rely on single-parameter metrics such as the Rate of Total Electron Content (TEC) Index (ROTI) or spatial TEC gradients considered independently, limiting their capability to characterize complex space–time ionospheric dynamics. We introduce a quantile-based composite ionospheric disturbance estimator designed for RTK positioning reliability assessment. The proposed framework integrates temporal ionospheric variability and spatial Vertical TEC (VTEC) gradient information into a unified risk indicator. Short-term ionospheric irregularities are characterized using rolling-median ROTI values, from which a high-quantile regional disturbance metric is extracted. Spatial ionospheric structure is quantified through interpolation of the VTEC field and computation of the gradient magnitude, followed by high-quantile extraction of gradient intensity. Both components are normalized using an adaptive quantile-based scaling scheme to ensure robustness against extreme values and regional statistical variability. The final RTK disturbance estimator is formulated as a weighted composite index combining normalized temporal and spatial disturbance measures. The method is fully reproducible and independent of absolute TEC magnitude, relying solely on GNSS-derived ionospheric observables. Validation using dense multi-frequency GNSS observations demonstrates that the composite estimator captures disturbance patterns not resolved by global ionospheric models and provides a physically interpretable risk score relevant for high-precision GNSS applications. The proposed approach offers a generalizable framework for ionospheric integrity monitoring and composite risk assessment in real-time GNSS positioning systems. The proposed approach provides a reproducible GNSS-based framework for composite disturbance monitoring. In this study, the method is validated using a mid-latitude regional CORS network (Latvia); extension to other ionospheric regimes and sparse networks requires further investigation.

## Introduction

The ionosphere remains one of the dominant error sources in high-precision Global Navigation Satellite System (GNSS) positioning, particularly for real-time kinematic (RTK) techniques that rely on rapid ambiguity resolution and carrier-phase consistency. Although dual-frequency observations effectively remove first-order ionospheric delay, short-term irregularities and spatial electron density gradients continue to degrade positioning reliability, increase convergence time, and compromise solution integrity. These effects are especially critical during disturbed ionospheric conditions, where localized gradients and rapid temporal fluctuations may not be adequately captured by conventional mitigation strategies.

Common ionospheric disturbance indicators, such as the Rate of Total Electron Content (TEC) Index (ROTI) or vertical TEC (VTEC) gradients, are typically analysed independently^1–4^. ROTI primarily characterizes short-term temporal variability along individual signal paths, whereas spatial TEC gradients describe horizontal electron density structure across a region. While both metrics provide valuable information, their separate use limits the ability to quantify the combined spatiotemporal disturbance state that directly affects RTK positioning performance. Moreover, most existing approaches rely on mean-based statistics or fixed empirical thresholds, which may lack robustness under highly variable ionospheric conditions.

The increasing availability of dense multi-GNSS reference station networks enables high-resolution monitoring of ionospheric structure using carrier-phase observations. However, transforming these observations into a physically interpretable and operationally meaningful positioning risk metric requires a unified and statistically robust formulation that integrates temporal and spatial disturbance characteristics within a single framework.

In this study, we introduce a quantile-based composite ionospheric disturbance estimator designed specifically for RTK positioning reliability assessment. The proposed method integrates high-quantile temporal ROTI metrics with high-quantile spatial VTEC gradient intensities derived from interpolated ionospheric fields. To ensure statistical robustness and independence from absolute TEC magnitude, both disturbance components are normalized using an adaptive quantile-based scaling scheme. The final estimator is formulated as a weighted composite index that reflects the coupled influence of temporal irregularities and spatial gradients on high-precision GNSS positioning.

Unlike regional case-specific modeling approaches, the proposed framework is reproducible and transferable in principle to other multi-GNSS networks, because it relies only on standard dual-frequency observables and robust quantile-based aggregation. However, the present manuscript evaluates the method only under mid-latitude conditions using the Latvian Continuously Operating Reference Station (CORS) network, and performance in other ionospheric regimes is left for future work. The methodology focuses on algorithmic formulation rather than regional calibration and is intended to support integrity monitoring, disturbance classification, and composite ionospheric risk assessment in real-time GNSS applications.

## Methods

### GNSS observation data and preprocessing

The proposed framework uses dual-frequency, multi-constellation GNSS observations collected from a dense CORS network. Observation data are processed in Receiver Independent Exchange (RINEX) format and include GPS and Galileo measurements with a 1 s sampling interval. Broadcast navigation messages are used to compute satellite positions and receiver–satellite geometry at each epoch.

Receiver coordinates are taken from the RINEX headers and assumed constant within a daily session. Satellite positions are computed in the Earth-centred Earth-fixed (ECEF) frame and transformed to a local topocentric frame to obtain elevation and azimuth angles. Observations below a minimum elevation threshold are excluded to reduce multipath and mitigate residual tropospheric and antenna effects. An elevation cutoff angle of 20$^\circ$ was applied for all carrier-phase observations. Strict ephemeris time-validity criteria are applied to ensure geometric consistency.

### Network spatial extent and quantile stability

The analysis is performed using observations from five GNSS reference stations distributed across the territory of Latvia. The network therefore spans the national-scale domain of approximately 300–400 km, covering mid-latitude ionospheric conditions typical for Northern Europe.

Using a regional aggregation increases the number of station–satellite observation links contributing to the high-quantile statistics at each epoch, which improves the stability of the estimated disturbance indicators. In larger spatial domains the quantile estimates become statistically more stable due to the larger number of samples. However, increasing the region size may partially smooth highly localized ionospheric structures. Conversely, very small networks may produce less stable quantile estimates due to the limited number of observation links. The selected domain therefore represents a compromise between spatial representativeness and statistical robustness for regional disturbance monitoring.

Station density primarily affects the stability of the spatial-gradient component, because gridded interpolation and numerical gradients become ill-conditioned when only few stations are available or when the spatial coverage is highly anisotropic. In sparse networks, the temporal component (ROTI-based aggregation) remains directly computable, while gradient estimation may require larger time bins, stronger regularization, or alternative pairwise baseline-gradient formulations.

### Slant TEC from the geometry-free dual-frequency carrier-phase combination

Dual-frequency carrier-phase observations are used to derive Slant Total Electron Content (STEC) due to their low measurement noise. The ionospheric contribution is isolated using the geometry-free (GF) linear combination of carrier phases^5,6^:

1 $$\begin{aligned} L_{\textrm{GF}} = \lambda _1 \Phi _1 - \lambda _2 \Phi _2 , \end{aligned}$$

where $\Phi _1$ and $\Phi _2$ are the carrier-phase observables at frequencies $f_1$ and $f_2$, and $\lambda _i$ denotes wavelength. STEC (in TECU) is obtained as

2 $$\begin{aligned} \textrm{STEC}_{\phi } = \frac{f_1^2 f_2^2}{40.3\,(f_1^2 - f_2^2)} \, L_{\textrm{GF}} . \end{aligned}$$

### Satellite OSB, receiver DCB estimation, smoothing, and phase leveling

To ensure unbiased ionospheric estimates, satellite Observable-Specific Bias (OSB) corrections are applied prior to TEC computation^7^. Receiver differential code biases (DCB) are estimated from the data using robust statistics. Code observations are smoothed using a Hatch filter to reduce measurement noise while preserving long-term behaviour.

Carrier-phase-derived STEC provides high precision but contains an unknown phase ambiguity term. Phase leveling aligns $\textrm{STEC}_{\phi }$ to code-derived STEC over continuous arcs, retaining phase precision while ensuring absolute consistency:

3 $$\begin{aligned} \textrm{STEC}_{\textrm{lev}} = \textrm{STEC}_{\phi } + \Delta _{\textrm{bias}}, \end{aligned}$$

where $\Delta _{\textrm{bias}}$ is estimated as the median difference between smoothed code and carrier-phase combinations within each continuous observation segment.

Cycle slips are detected and removed using geometry-free and Melbourne–Wübbena combinations, preventing discontinuities from propagating into the TEC time series.

### Vertical TEC mapping with a single-layer ionospheric model

VTEC is obtained from leveled STEC using a single-layer ionospheric model (SLM)^8^. The mapping from slant to vertical TEC is given by

4 $$\begin{aligned} \textrm{VTEC} = \textrm{STEC}_{\textrm{lev}} \cdot M^{-1}(e), \end{aligned}$$

where *e* is satellite elevation and

5 $$\begin{aligned} M(e) = \left( 1 - \left( \frac{R_{\textrm{E}} \cos e}{R_{\textrm{E}} + H_{\textrm{ion}}} \right) ^2 \right) ^{-1/2}. \end{aligned}$$

Here $R_{\textrm{E}}$ is the mean Earth radius and $H_{\textrm{ion}}=450$ km is the effective ionospheric shell height. Sensitivity to reasonable variations of shell height does not significantly affect the high-quantile disturbance extraction used in this study. This choice is consistent with common mid-latitude modeling practice.

Ionospheric pierce points (IPP) are computed for each observation epoch assuming a spherical Earth and the fixed shell height. No spatial averaging is applied at this stage, preserving the native spatiotemporal resolution of the network for subsequent analysis.

### ROTI computation and temporal disturbance extraction

Short-term ionospheric variability is quantified using the ROTI, computed from the time derivative of STEC within a sliding window^1,9,10^:

6 $$\begin{aligned} \textrm{ROTI} = \sqrt{ \left\langle \left( \frac{\Delta \textrm{STEC}}{\Delta t} \right) ^2 \right\rangle - \left\langle \frac{\Delta \textrm{STEC}}{\Delta t} \right\rangle ^2 } , \end{aligned}$$

where $\Delta t$ is the sampling interval and $\langle \cdot \rangle$ denotes averaging over the window.

To reduce sensitivity to short-lived spikes while preserving disturbed conditions, ROTI values are aggregated using a rolling-window median per station–satellite arc (window length 120 s). A network-level temporal disturbance metric is then defined at each epoch *t* as the high-quantile across all station–satellite combinations:

7 $$\begin{aligned} R_{\textrm{ROTI}}(t) = Q_{0.95}\left( \textrm{ROTI}(t)\right) . \end{aligned}$$

The disturbance metric is computed at the regional network level rather than for a single station. For each epoch, ROTI values are aggregated across all available station–satellite links within the considered CORS network, and the high quantile is extracted from this combined distribution.

In this study the regional domain corresponds to the Latvian CORS network, covering approximately 300–400 km in horizontal extent with typical inter-station spacing of 40–70 km. This spatial scale is sufficiently large to capture regional ionospheric disturbance structure while maintaining statistical robustness of the quantile estimator. For dense networks with multiple simultaneous station–satellite observations, the high-quantile statistic remains stable even when individual links are affected by local measurement noise or temporary signal loss.

### Spatial VTEC gradient intensity from gridded interpolation

Spatial gradients of TEC/VTEC derived from ground-based GNSS observations are widely used to characterize ionospheric structure and disturbance dynamics^2^. Recent approaches further separate spatial and temporal gradient components to better interpret multi-scale ionospheric variability^3,4^. Spatial ionospheric structure is characterized by VTEC gradients derived from the station-level VTEC time series. For each epoch, station VTEC values are aggregated within fixed time bins (120 s) using the median per station to increase robustness against outliers.

Station coordinates are transformed from ECEF to geodetic latitude and longitude. For each time bin, VTEC values are interpolated onto a regular latitude–longitude grid using linear interpolation. The spatial gradient magnitude is then computed on the grid as

8 $$\begin{aligned} |\nabla \textrm{VTEC}(t)| = \sqrt{ \left( \frac{\partial \textrm{VTEC}}{\partial x} \right) ^2 + \left( \frac{\partial \textrm{VTEC}}{\partial y} \right) ^2 }. \end{aligned}$$

Grid spacing is converted to approximate distances in kilometres using 111.32 km per degree of latitude and $111.32\cos (\varphi )$ km per degree of longitude at the mean grid latitude $\varphi$.

A regional spatial disturbance metric is extracted as the high-quantile of the gradient magnitude field:

9 $$\begin{aligned} R_{\textrm{GRAD}}(t) = Q_{0.95}\left( |\nabla \textrm{VTEC}(t)|\right) . \end{aligned}$$

If an epoch does not provide sufficient stations for stable interpolation, it is omitted; the resulting $R_{\textrm{GRAD}}(t)$ time series is forward-filled to avoid artificial gaps in the composite estimator.

The 95th percentile was selected as a compromise between sensitivity to elevated disturbance conditions and statistical stability of the regional aggregation. During method development, several alternative quantile thresholds (Q$_{0.75}$, Q$_{0.85}$, Q$_{0.90}$ and Q$_{0.99}$) were evaluated. Lower quantiles produced smoother time series and tended to suppress localized disturbance peaks, while very high quantiles increased sensitivity to rare extreme values and introduced higher epoch-to-epoch variability. The Q$_{0.95}$ threshold therefore preserves elevated disturbance levels while remaining robust against individual observation-link outliers. A qualitative sensitivity check indicated that the main disturbance periods and the overall association trends remain consistent across the tested quantiles, while Q$_{0.95}$ provides the best balance of interpretability and stability for operational monitoring.

### Adaptive quantile-based normalization

To ensure robustness and comparability across varying ionospheric backgrounds, both disturbance components are normalized using adaptive quantile scaling. For a time series *X*(*t*), the normalized form is defined as

10 $$\begin{aligned} \tilde{X}(t) = \frac{X(t) - Q_{0.05}(X)}{Q_{0.95}(X) - Q_{0.05}(X)}. \end{aligned}$$

If $Q_{0.95}(X)\le Q_{0.05}(X)$, $\tilde{X}(t)$ is set to zero for numerical stability. The use of quantile-based normalization ensures robustness against extreme values and heavy-tailed distributions^11,12^.

### Composite indicator definition

The final composite RTK disturbance estimator is formulated as

11 $$\begin{aligned} \mathrm {RTK\_RISK}(t) = w_1 \tilde{R}_{\textrm{ROTI}}(t) + w_2 \tilde{R}_{\textrm{GRAD}}(t), \end{aligned}$$

where $w_1$ and $w_2$ are weighting coefficients controlling the relative influence of temporal irregularities and spatial gradients. In this study equal weights ($w_1 = w_2$) are adopted in order to provide a balanced representation of temporal ionospheric variability and spatial electron density gradients. This formulation yields a dimensionless risk indicator reflecting the coupled spatiotemporal ionospheric state relevant for RTK positioning reliability. For visualization, the RTK_RISK time series is optionally smoothed using a rolling mean with a 120 s window and displayed with low/medium/high risk zones.

### Risk level classification

For visualization and operational interpretation in Fig. 3, the smoothed $\mathrm {RTK\_RISK}$ time series is classified into three disturbance levels using empirical quantiles of the daily $\mathrm {RTK\_RISK}$ distribution.

Risk levels are defined using the 50th percentile ($Q_{0.50}$) and the 80th percentile ($Q_{0.80}$) of the $\mathrm {RTK\_RISK}$ distribution. Low risk corresponds to values below $Q_{0.50}$, medium risk to values between $Q_{0.50}$ and $Q_{0.80}$, and high risk to values above $Q_{0.80}$.

A quantile-based approach was selected because the RTK_RISK index is a composite indicator derived from ionospheric variability metrics and its distribution is typically non-Gaussian with occasional extreme values during disturbed ionospheric conditions. Quantile thresholds therefore provide a robust and data-driven method for defining relative disturbance levels without relying on fixed empirical limits.

Under this classification, the high-risk category corresponds to the upper 20% of disturbance conditions observed in the analysed dataset.

### Proxy validation using kinematic baseline processing

To provide a minimal link between the proposed ionospheric disturbance estimator and ambiguity resolution behaviour, we performed a kinematic baseline processing experiment using RTKLIB. Dual-frequency GPS+Galileo observations from two CORS stations (LGIA as reference and LVR1 as rover; baseline length $\sim$50 km) were processed in kinematic mode, yielding epoch-wise solution status (fixed/float), the ambiguity ratio-test statistic, and horizontal position error with respect to the known rover coordinates. These quantities are standard ambiguity-resolution performance proxies and enable a quantitative association analysis with the proposed $\mathrm {RTK\_RISK}$ indicator under the same ionospheric conditions.

## Results

### Validation against global ionospheric models

Independent validation of locally derived VTEC estimates is performed using multiple Global Ionosphere Map (GIM) products provided in IONEX format by international analysis centres, including CODE, ESA, IGS, and JPL^13,14^. These global products are not used within the local estimation procedure.

For each GIM product, the VTEC grid is interpolated spatially and temporally to the IPP locations and observation epochs of the local dataset. This enables direct point-by-point comparison without enforcing agreement through bias alignment or tuning.

**Fig. 1:**
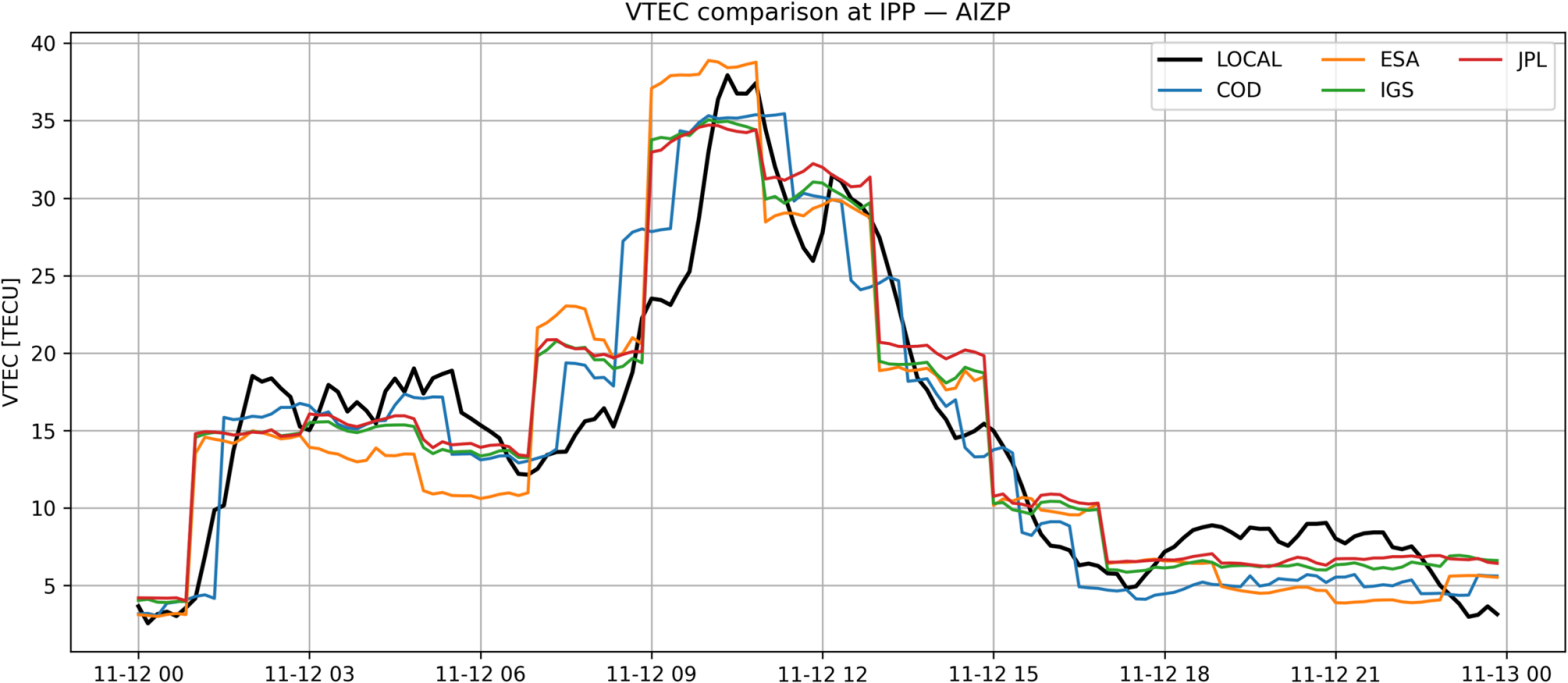
Comparison of locally derived VTEC and Global Ionosphere Map products (CODE, ESA, IGS, JPL) at the ionospheric pierce point for CORS station AIZP.

Figure 1 presents a representative comparison between locally derived VTEC and selected GIM products at the AIZP station. All global models reproduce the large-scale diurnal trend, while regional GNSS observations resolve additional short-term variability that is partially smoothed in global products.

To further quantify systematic deviations, VTEC differences are computed at the observation level and aggregated using robust statistics (bias, RMSE, MAE, and correlation). Table 1 summarizes the statistical agreement between the regional solution and the considered GIM products.

Although global ionospheric models reproduce the dominant diurnal TEC variation, the observed RMSE values (12–13 TECU) indicate substantial local deviations at high temporal resolution. Correlation coefficients (r - 0.55–0.57) confirm moderate agreement at large scales, but also demonstrate that short-term and regional-scale variability is only partially captured by global products.

These unresolved local fluctuations directly affect ambiguity resolution in RTK positioning, where even moderate ionospheric gradients may introduce centimeter-level positioning errors or delay integer ambiguity fixing. The composite RTK_RISK estimator is therefore designed to capture precisely this high-resolution disturbance component that is smoothed in global ionospheric maps.

**Table 1:** Statistical comparison between locally derived VTEC and Global Ionosphere Map (GIM) products at station AIZP.

Model	Bias (TECU)	RMSE (TECU)	MAE (TECU)	*r*	*N*
CODE	$-4.93$	12.72	11.43	0.570	1096923
ESA	$-5.36$	13.32	11.79	0.542	1096923
IGS	$-5.70$	13.11	11.61	0.556	1096923
JPL	$-6.23$	13.36	11.75	0.555	1096923

Bias denotes mean difference (GIM minus local VTEC). *RMSE* root-mean-square error, *MAE* mean absolute error, *r* Pearson correlation coefficient, *N* number of compared samples.

**Fig. 2:**
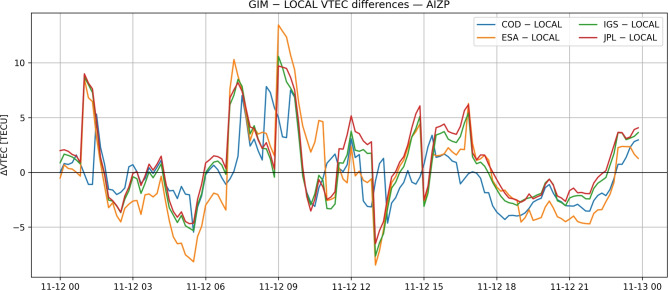
Temporal evolution of VTEC differences between global ionospheric models (CODE, ESA, IGS, JPL) and locally derived VTEC at station AIZP.

Figure 2 illustrates the temporal evolution of VTEC differences between global ionospheric models and locally derived estimates at station AIZP. The increased dispersion during periods of enhanced ionospheric activity reflects resolution-dependent smoothing effects in global models.

**Fig. 3:**
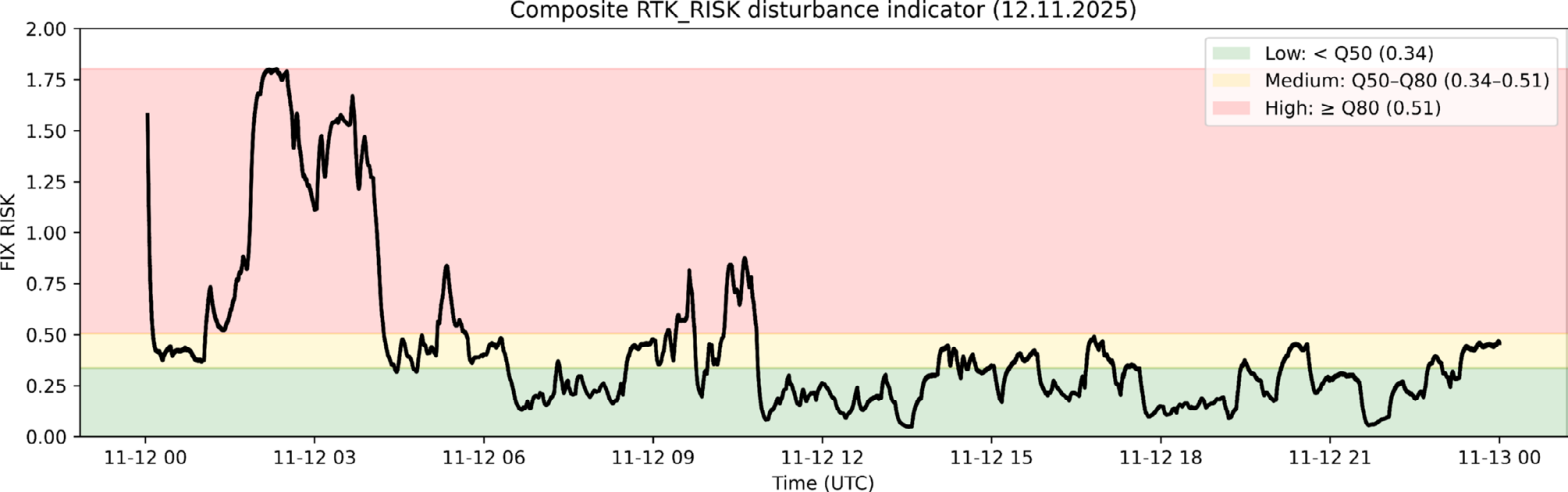
Composite $\mathrm {RTK\_RISK}$ time series on 12 November 2025. Shaded regions indicate operational disturbance levels derived from empirical quantiles of the RTK_RISK distribution (low: $<Q_{0.50}$, medium: $Q_{0.50}$–$Q_{0.80}$, high: $\ge Q_{0.80}$).

Figure 3 shows the composite $\mathrm {RTK\_RISK}$ time series derived from the proposed quantile-based estimator. Elevated values correspond to periods characterized by simultaneous high temporal ROTI variability and strong spatial VTEC gradients.

To evaluate the operational relevance of the proposed estimator, we analysed the statistical association between the disturbance indicators and positioning performance metrics derived from kinematic baseline processing.

Spearman correlation analysis indicates a statistically significant association between $\mathrm {RTK\_RISK}$ and horizontal positioning error ($\rho = 0.27$, $p < 10^{-16}$). Similarly, the temporal irregularity indicator ROTI shows a moderate positive correlation with positioning error ($\rho = 0.34$, $p < 10^{-16}$).

The ambiguity ratio exhibits a negative correlation with $\mathrm {RTK\_RISK}$ ($\rho = -0.26$, $p < 10^{-16}$), indicating decreasing ambiguity resolution quality during disturbed ionospheric conditions.

Comparison with individual disturbance indicators shows that ROTI alone provides a strong association with positioning error, whereas spatial TEC gradient intensity exhibits a weaker relationship. The composite $\mathrm {RTK\_RISK}$ estimator combines temporal and spatial disturbance components within a unified framework and therefore provides a more general disturbance indicator that remains sensitive to both temporal irregularities and spatial ionospheric structure. These results indicate that while ROTI captures strong temporal ionospheric variability, the composite RTK_RISK indicator provides a physically interpretable disturbance metric integrating both temporal and spatial ionospheric effects relevant for RTK ambiguity resolution.

### Association with ambiguity resolution and positioning error (proxy experiment)

**Fig. 4:**
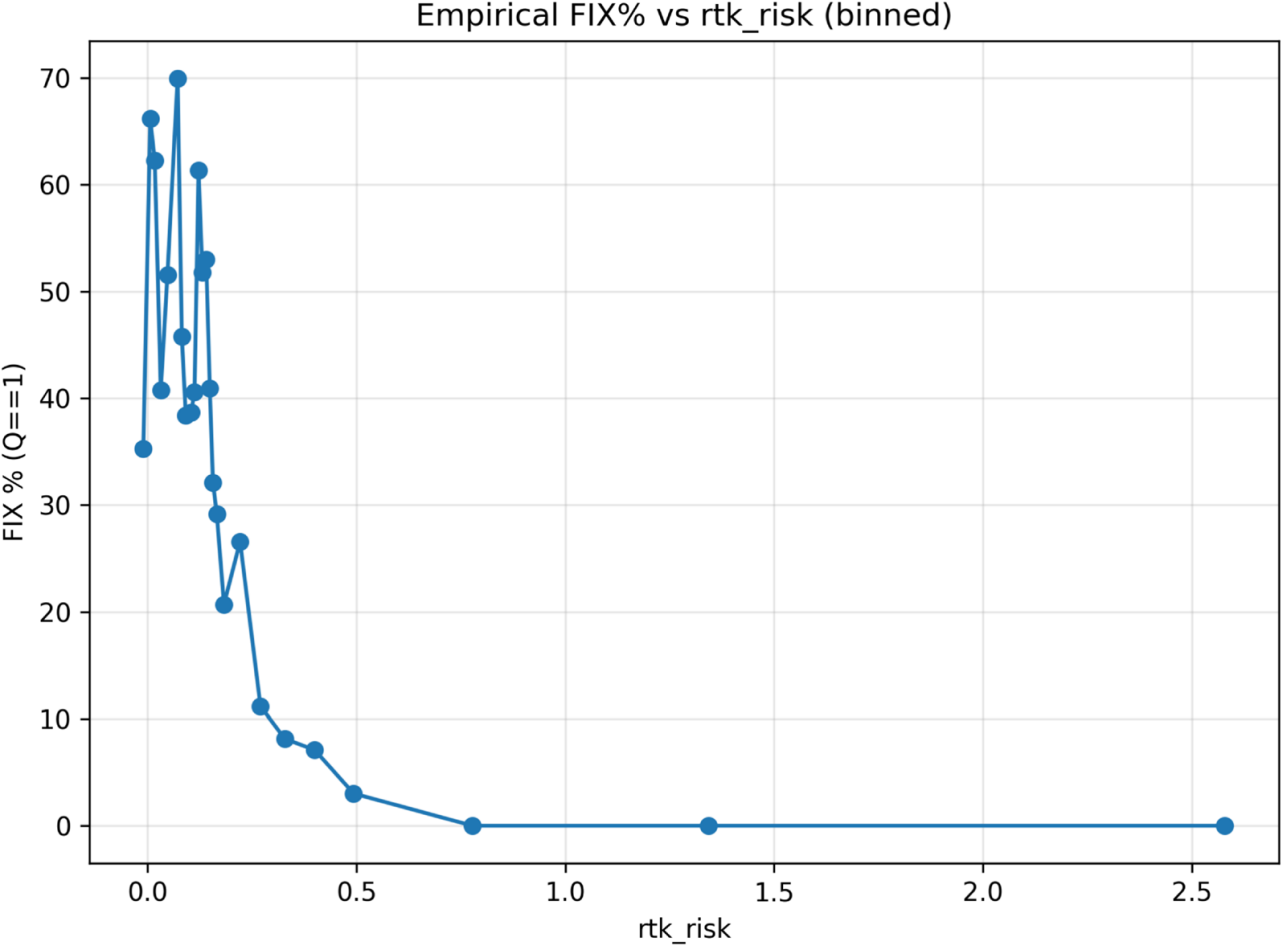
Empirical FIX availability as a function of the composite $\mathrm {RTK\_RISK}$ disturbance level. The FIX percentage decreases with increasing disturbance levels, indicating reduced ambiguity resolution reliability during ionospheric disturbances.

Figure 4 shows the empirical FIX% as a function of $\mathrm {RTK\_RISK}$, indicating a clear decrease in FIX availability with increasing disturbance levels.

To provide an operational proxy validation, the empirical availability of fixed ambiguity solutions was analysed as a function of the composite disturbance indicator.

Figure 4 shows the empirical FIX percentage obtained from kinematic baseline processing as a function of $\mathrm {RTK\_RISK}$. A clear decrease in FIX availability is observed with increasing disturbance levels. When the RTK_RISK indicator exceeds approximately 0.3–0.4, the FIX rate drops rapidly and approaches zero at high disturbance levels.

This behaviour confirms that elevated ionospheric disturbance conditions captured by the composite estimator are associated with degraded ambiguity resolution performance.

In addition, correlation and group-difference statistics (Tables 2 and 3) show that horizontal positioning error increases and ambiguity resolution quality decreases under higher disturbance levels.

Table 2 reports Pearson and Spearman correlations between the proposed composite disturbance indicator ($\mathrm {RTK\_RISK}$), its individual components (ROTI-only temporal irregularity and gradient-only spatial disturbance metrics), and the kinematic ambiguity-resolution proxies derived from RTKLIB processing (horizontal error and ambiguity ratio-test statistic).

ROTI-only shows the strongest association with horizontal positioning error (Spearman $\rho = 0.337$), indicating that rapid temporal ionospheric variability primarily affects positioning accuracy. In contrast, the gradient-only metric exhibits a clearer association with the ambiguity ratio statistic (Spearman $\rho = 0.220$) and a weaker relationship with horizontal error, reflecting the influence of spatial TEC gradients on ambiguity resolution stability.

The composite $\mathrm {RTK\_RISK}$ indicator remains significantly associated with both horizontal error (Spearman $\rho = 0.265$) and ambiguity ratio (Spearman $\rho = -0.265$), indicating that the combined metric captures both temporal irregularities and spatial ionospheric structure simultaneously.

**Table 2:** Correlation between ionospheric disturbance indicators and kinematic ambiguity-resolution/positioning metrics from RTKLIB processing (LGIA–LVR1, $\sim$50 km). Pearson measures linear association and Spearman measures monotonic association.

Pair	*N*	Pearson *r*	*p*	Spearman $\rho$	*p*
RTK_RISK vs err_h_m	82829	0.3490545	$p < 0.001$	0.265	$p < 0.001$
ROTI vs err_h_m	82829	0.3683946	$p < 0.001$	0.337	$p < 0.001$
GRAD vs ratio	82829	0.3464728	$p < 0.001$	0.220	$p < 0.001$
GRAD vs err_h_m	82829	0.0150295	$p = 0.0000152$	$-0.170$	$p < 0.001$
RTK_RISK vs ratio	82829	0.0000000	$p < 0.001$	$-0.265$	$p < 0.001$

**Table 3:** Summary statistics for ambiguity-fixed (Q=1) and float (Q=2) epochs in the kinematic baseline experiment.

Metric	Group	*N*	Median	IQR	Mean ± Std
RTK_RISK	FIXED (Q=1)	30161	0.121	0.142	0.343 ± 0.647
RTK_RISK	FLOAT (Q=2)	52668	0.266	0.635	0.802 ± 1.854
err_h_m	FIXED (Q=1)	30161	0.048	0.052	0.065 ± 0.121
err_h_m	FLOAT (Q=2)	52668	0.512	0.977	0.955 ± 1.268
err_3d_m	FIXED (Q=1)	30161	0.077	0.073	0.123 ± 0.378
err_3d_m	FLOAT (Q=2)	52668	0.732	1.367	1.439 ± 1.872
ratio	FIXED (Q=1)	30161	3.7	1.4	4.78 ± 3.11
ratio	FLOAT (Q=2)	52668	1.1	0.2	1.20 ± 0.31

Median and interquartile range (IQR) describe robust central tendency, while mean and standard deviation (Std) are shown for completeness.

Non-parametric Mann–Whitney tests confirmed statistically significant differences between FIXED and FLOAT epochs for $\mathrm {RTK\_RISK}$ and horizontal error ($p<0.05$), with large effect sizes (Cliff’s $\delta$), indicating substantially degraded ambiguity resolution during high-disturbance conditions.

### Relationship between RTK_RISK and RTK positioning performance

When applied to the full network, the composite RTK_RISK estimator exhibits elevated values during periods of enhanced temporal variability and strong spatial gradients, demonstrating its capability to capture disturbance states relevant for positioning integrity.

To quantify the relationship between ionospheric disturbance indicators and RTK positioning performance, correlation analysis was performed between disturbance metrics and positioning quality indicators derived from RTKLIB solutions.

Spearman correlation analysis indicates a statistically significant association between RTK_RISK and horizontal positioning error ($\rho = 0.27$, $p < 10^{-16}$). The temporal irregularity indicator ROTI shows a stronger correlation with positioning error ($\rho = 0.34$, $p < 10^{-16}$), while the spatial TEC gradient metric exhibits a clearer association with the ambiguity ratio statistic ($\rho = 0.22$, $p < 10^{-16}$).

These results indicate that temporal ionospheric variability primarily affects positioning accuracy, whereas spatial gradients are more closely associated with ambiguity resolution degradation. The composite RTK_RISK indicator captures both disturbance mechanisms simultaneously.

## Discussion

Traditional ionospheric monitoring approaches often rely on either temporal irregularity indicators such as ROTI or spatial gradient analysis considered independently. The presented composite formulation integrates both components within a statistically robust quantile-based framework.

The use of high-percentile extraction suppresses isolated outliers while preserving elevated disturbance conditions that are most relevant for RTK ambiguity resolution. Adaptive quantile normalization further improves robustness to heavy-tailed distributions and reduces dependence on absolute TEC magnitude and seasonal background variability.

Comparison with global ionospheric models highlights that dense GNSS observations resolve regional structures partially smoothed in coarse-resolution products. The composite estimator therefore provides a physically interpretable disturbance metric that is suitable for integrity-oriented monitoring and operational disturbance classification in high-precision GNSS applications^15^.

The statistical proxy experiment indicates that the temporal irregularity component (ROTI) shows the strongest association with horizontal positioning error, whereas the spatial TEC-gradient component exhibits a clearer relationship with the ambiguity ratio statistic. The composite $\mathrm {RTK\_RISK}$ indicator captures both disturbance mechanisms simultaneously, supporting its use as a unified regional disturbance descriptor.

Finally, we note important applicability constraints. The current validation is performed for a mid-latitude regional network (Latvia). In low-latitude equatorial anomaly regions and high-latitude auroral zones, disturbances may exhibit different spatial scales and temporal morphology; therefore, the empirical mapping between $\mathrm {RTK\_RISK}$ levels and RTK performance may differ and requires dedicated validation using representative datasets. In addition, the method is most reliable in dense networks because the spatial VTEC-gradient component depends on multi-station interpolation^16,17^. In sparse networks, the ROTI-based temporal component remains directly computable, whereas gradient estimation may become unstable and may require simplified pairwise baseline-gradient formulations, stronger regularization, or coarser time/space resolution.

Although $\mathrm {RTK\_RISK}$ is designed to represent ionospheric conditions relevant for RTK ambiguity resolution, the present study does not calibrate a direct failure probability model (e.g., FIX rate or time-to-first-fix) across different receivers and network RTK implementations. Therefore, the index should be interpreted as a proxy indicator of ionospheric disturbance conditions potentially affecting RTK reliability.

## Data Availability

GNSS observation data used in this study were obtained from the Latvian permanent GNSS network (LatPos). Dual-frequency multi-constellation RINEX (v3.04) observation files with 1 s sampling interval were used in the analysis. RINEX data are publicly available free of charge; access requires prior user registration and account approval via the official data request interface: https://latpos.lgia.gov.lv/SBC/User/Xpos/RinexDataRequest. Satellite Observable-Specific Bias (OSB) corrections were obtained from the Center for Orbit Determination in Europe (CODE) archive. Daily final OSB bias products were accessed from: http://ftp.aiub.unibe.ch/CODE/. Global Ionosphere Map (GIM) products in IONEX format (CODE, ESA, IGS, and JPL analysis centers) used for validation were accessed from the NASA CDDIS archive: https://cddis.nasa.gov/archive/gnss/products/ionex/. Access to CDDIS data requires free NASA Earthdata user registration for authentication: https://urs.earthdata.nasa.gov. No proprietary datasets were used in this study.

## Code availability

The processing workflow was implemented in Python (version 3.11) using standard scientific libraries including NumPy, Pandas, matplotlib and SciPy. All parameter settings required for reproducing the composite estimator are explicitly provided in the Methods section. The source code is not publicly available due to ongoing commercialization and intellectual property development. All computations were performed using double-precision floating-point arithmetic.
